# Dendrimer Nanogels with Built-in Free Radical Scavenging Enable Efficient Topical Delivery of a Hydrophilic Antioxidant to Restore Lens Redox Balance for Cataract Treatment

**DOI:** 10.1021/acsami.5c24072

**Published:** 2026-03-19

**Authors:** Lin Qi, Huari Kou, Anna Chernatynskaya, Da Huang, Vimalin Jeyalatha Mani, Humeyra Karacal, Nuran Ercal, Hu Yang

**Affiliations:** † Joint Department of Biomedical Engineering, 5506 Marquette University and Medical College of Wisconsin, Milwaukee, Wisconsin 53226, United States; ‡ Linda and Bipin Doshi Department of Chemical and Biochemical Engineering, 14717 Missouri University of Science and Technology, Rolla, Missouri 65409, United States; § College of Biological Science and Engineering, 12423 Fuzhou University, Fuzhou, Fujian 350108, China; ∥ Premiere Eye Associates, 816 S. Kirkwood, St. Louis, Missouri 63122, United States; ⊥ Department of Chemistry, 14717 Missouri University of Science and Technology, Rolla, Missouri 65409, United States

**Keywords:** dendrimer-based nanogels, ocular drug delivery, N-acetylcysteine, ROS scavenging, cataract

## Abstract

Cataract is a leading cause of vision impairment worldwide and are primarily caused by oxidative stress that damages and aggregates lens proteins, leading to lens opacification. However, the eye’s anatomical barriers limit the penetration and bioavailability of antioxidant therapies. To address this challenge, a dendrimer-based nanogel with a built-in reactive oxygen species (ROS)-scavenging capability developed by us was employed to deliver the antioxidant N-acetylcysteine (NAC) to the lens. NAC was loaded into a generation-5 PEGylated poly­(amidoamine) dendrimer (G5-PEG-TK, termed the GPT) nanogel. The resulting NAC-GPT was characterized for its ROS-scavenging activity, bioavailability, and corneal permeability. The efficacy of NAC-GPT was evaluated *ex vivo* and *in vivo* using a sodium selenite (Na_2_SeO_3_)-induced cataract model. Both *ex vivo* and *in vivo* results demonstrated that NAC-GPT significantly increased the level of NAC accumulation in the lens. Furthermore, the *in vivo* study shows that NAC-GPT significantly increased the ratio of reduced glutathione (GSH) to oxidized glutathione (GSSG), an indicator of redox balance restoration. In particular, the GSH/GSSG ratio in NAC-GPT–treated lenses was nearly 2-fold higher than that of the untreated cataract control. These findings indicate that the GPT nanogel platform can effectively deliver antioxidants to the eye and is a promising noninvasive antioxidant delivery strategy with the ability to restore redox balance in cataract.

## Graphical abstract

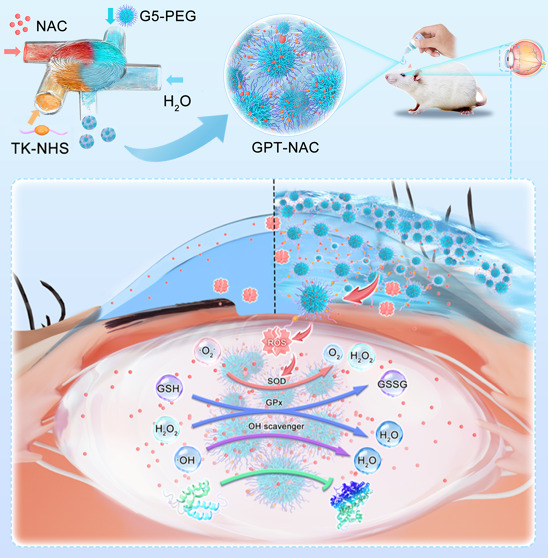

## Introduction

Cataract, characterized by lens clouding, remains the leading cause of blindness worldwide, accounting for more than 50% of all eye diseases.^1^ Currently, surgical replacement of an intraocular lens (IOL) is the main treatment for cataracts, but it carries risks such as IOL opacification, capsular rupture, vitreous loss, and endophthalmitis.^2,3^ Moreover, the natural lens has several irreplaceable functions. It is capable of dynamic stretching for accommodation, allowing the eye to focus on objects at varying distances, and it also plays a key role in maintaining ocular antioxidant defense by acting as a reservoir for GSH.^4^ Loss of GSH supply following lens removal may increase the risk of oxidative stress–related ocular disorders, such as glaucoma and macular degeneration.^5,6^ Therefore, preventing or delaying lens opacification, especially in the early stages of cataract development, is of critical importance.

Despite advancements in medical science, there are currently no medications specifically designed for the prevention or treatment of cataracts. Studies indicate that oxidative stress is the primary contributor to cataract formation.^7^ It is caused by the imbalance between ROS production and depletion of innate neutralization system in the body.^8^ This imbalance leads to the production of oxidants at a rate that far exceeds the ability of the body to counteract them. Nevertheless, certain supplements and antioxidant drugs have demonstrated potential in slowing the progression of cataracts. While naturally occurring antioxidants such as GSH in the human body have gained attention, it is not sufficiently bioavailable and ineffective when given topically or orally.^9^ Similar limitations exist for other antioxidant, including vitamin C and vitamin E.^10^

One promising approach has been reported involving the use of eye drops containing NAC, a precursor to GSH.^11^ NAC demonstrates clinical potential in slowing cataract progression by increasing GSH levels in the lens, thereby reducing oxidative damage to lens proteins.^12^ However, the anatomical location of the lens in the deepest part of the anterior chamber of the eye poses significant challenges for effective drug delivery.^13^ Eye drops administered topically encounter several physiological barriers, including the tear film, corneal epithelium, and aqueous humor, and must subsequently traverse the lens capsule and intralenticular diffusion barriers to reach the lens nucleus.^14,15^ Additionally, NAC is a water-soluble small molecule that is rapidly metabolized and removed by tear flushing, resulting in a bioavailability of less than 1%.^16^ This severely limits NAC’s ability to reach therapeutic levels in the lens, hindering its effectiveness in preventing or slowing cataract progression. These limitations highlight the demands for noninvasive treatments and prevention strategies that can effectively deliver antioxidants, reduce oxidative stress, and restore redox balance in the lens.

Nanotechnology-based ocular drug delivery systems have gained significant attention for their efficient drug delivery capability compared with conventional drug delivery methods.^17,18^ Among the various nanotechnology platforms, functionalized nanogels have emerged as an effective and versatile drug delivery system due to their unique properties, such as high drug-loading capacity, controlled and sustained drug release, and excellent biocompatibility.^19^ In our previous work, we developed a G5-based ROS-scavenging nanogel system, termed G5-TK, for potential application in ocular drug delivery.^20^ To efficiently encapsulate the therapeutic agent within the nanogel matrix during rapid nanoprecipitation, we used a previously established flash nanoprecipitation technique that enables concurrent in situ cross-linking by replacing one aqueous inlet with a drug-containing solution.^21^ The drug-loading and cross-linking reactions are simultaneously achieved in this process. Different from our previous work, a hydrophilic drug, NAC, is loaded on this nanoparticle with a high loading capacity. Flash nanoprecipitation is initially designed for hydrophobic component nanoparticle synthesis. In the present study, this limit is overcome to construct hydrophilic drug and dendrimer nanoparticles (Figure 1).

**Figure 1:**
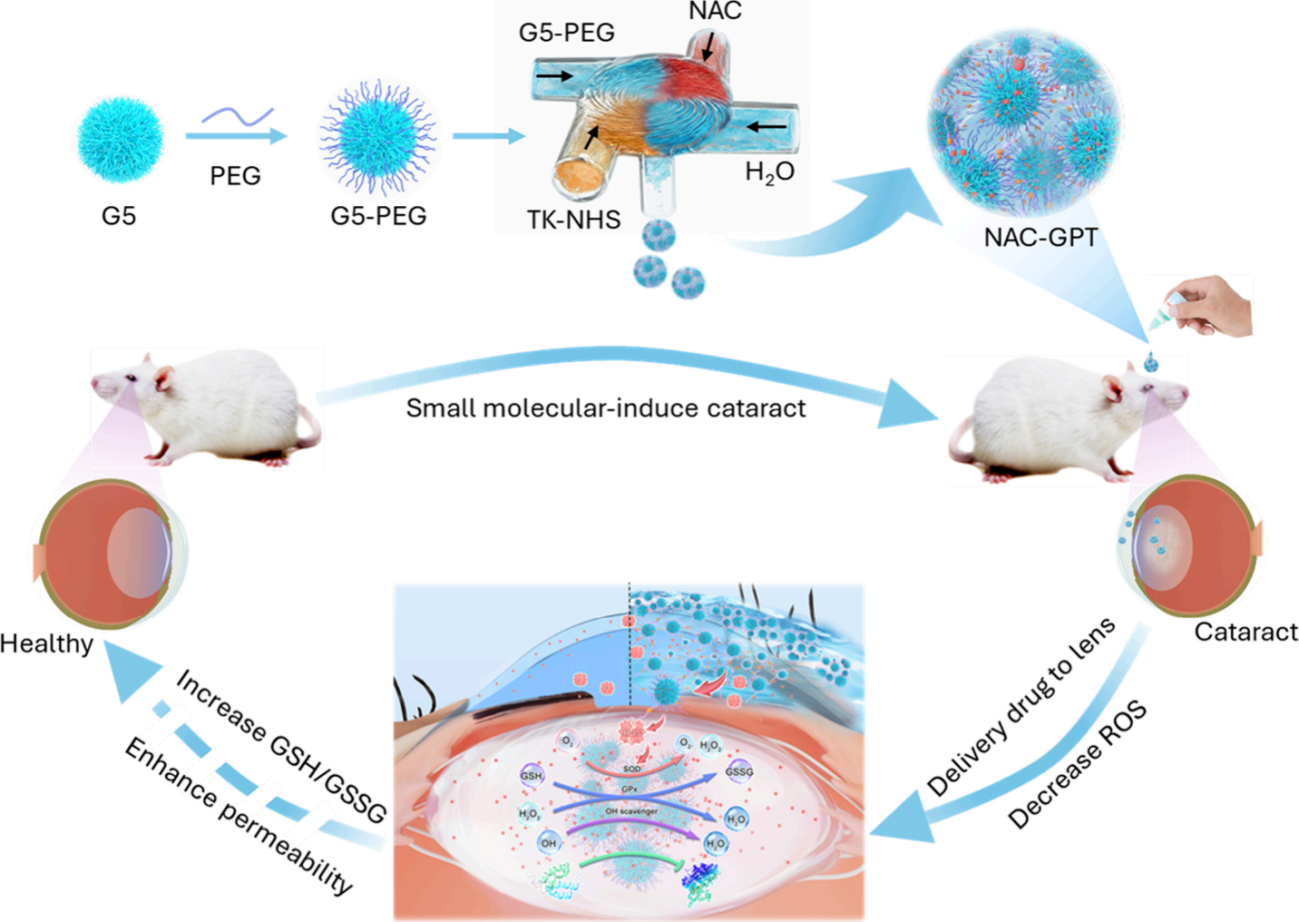
Illustration of ROS-responsive NAC-GPT nanogel formation, where the TK linker is conjugated to G5-PEG via MIVM while simultaneously loading the hydrophilic antioxidant drug NAC. The nanogels enhance corneal penetration, deliver NAC into the lens, and reduce intraocular ROS.

This study builds upon previous efforts to develop dendrimer-based nanogels with ROS-scavenging capabilities, optimizing the formulation to improve the stability, drug loading efficiency, and corneal permeation properties. The therapeutic potential of nanogel system was systematically evaluated through *ex vivo* and *in vivo* experiments, demonstrating its efficacy in delivering NAC to the lens, mitigating oxidative damage, and restoring redox homeostasis (Figure 1). The findings of this work demonstrate the promise of functionalized nanogels as an advanced platform for cataract treatment, providing efficient antioxidant delivery and enhanced ability to scavenge ROS.

## Experimental Section

### Materials and Characterization

EDA-core PAMAM dendrimer generation 5 (G5) was purchased from Dendritech (Midland, MI, USA). 1-Ethyl-3-(3-(dimethylamino)­propyl) carbodiimide (EDCI) was purchased from TCI America. IRDye 800CW NHS ester (800 CW-NHS) was from LICORbio-U.S. 3.4 kDa. PEG with NHS ester (PEG-NHS) was purchased from Ruixi (Xi’an, China). Sodium selenite (Na_2_SeO_3_, ≥99% pure), GSH, NAC, and GSSG were purchased from Sigma-Aldrich (St. Louis, MO, USA).

### Synthesis of G5-PEG-TK (GPT)

PAMAM G5 was dissolved in a 0.2 mM NaHCO_3_ aqueous solution with a concentration of 10 mg/mL. PEG-NHS was dissolved in DI water with a concentration of 11.7 mg/mL. Then, equal volumes of both solutions were mixed and stirred for 12 h at room temperature, allowing the reaction between *N*-hydroxysuccinimide ester and amine group.^22^ The final solution was purified by dialyzing against DI water (3.5 kDa molecular weight cutoff). The DI water was replaced with fresh DI water three times every 12 h. G5-PEG (GP) can be obtained as a white powder after freeze-drying. The chemical structures of GP and GPT in deuterium oxide (D_2_O) were confirmed using proton nuclear magnetic resonance spectroscopy (^1^H NMR) (Avance III 500 MHz, Bruker, Billerica, MA, USA).

### Synthesis of NAC-Loaded G5-PEG-TK (NAC-GPT)

NAC-GPT was cross-linked via the flash nanoprecipitation method. GP was dissolved in DI water at a concentration of 1 mg/mL. TK-NHS, a thioketal with *N*-hydroxysuccinimide (NHS) ester groups, was dissolved in acetone (0.3 mg/mL). NAC was dissolved in water with different concentrations from 0.4 to 1.2 mg/mL. A four-inlet vortex mixer was designed and used to synthesize nanoparticles as reported in our previous work.^21,23^ Two inlets separately contained GP (1 mg/mL) aqueous solution and TK-NHS (0.3 mg/mL) acetone solution, another was NAC (0.8 mg/mL), and the last channel was used for DI water. All these four solutions were pumped into a multi-inlet vortex mixer (MIVM) at a speed of 40 mL/min. The product solution was processed with an ultra-15 centrifugal filter (Millipore Sigma, Cat. No: UFC901024) to remove acetone and the free drug. NAC-GPT could be obtained after freeze-drying. To determine encapsulation efficiency (EE%) and loading capacity (LC%) of products, the samples were further tested with liquid chromatography–mass spectrometry (LC-MS). The EE% (eq 1) and LC% (eq 2) were calculated using the following formulas:

$$\mathrm{Encapsulation}\;\mathrm{efficiency}\;(\mathrm{EE}\%)=\frac{\mathrm{total}\;\mathrm{drug}\;\mathrm{added}-\mathrm{free}\;\mathrm{non}\;\mathrm{entrapped}\;\mathrm{drug}}{\mathrm{total}\;\mathrm{drug}\;\mathrm{added}}\times 100\%$$ 1

$$\mathrm{Loading}\;\mathrm{capacity}\;(\mathrm{LC}\%)=\frac{\mathrm{total}\;\mathrm{entrapped}\;\mathrm{drug}}{\mathrm{total}\;\mathrm{nano}\mathrm{particle}\;\mathrm{weight}}\times 100\%$$ 2

### *Ex Vivo* Whole Lens Incubation

Three-week-old male Sprague–Dawley (SD) rats were purchased from Charles River Laboratories (Wilmington, MA, USA). After a quarantine period, lenses were carefully extracted from whole eyeballs using a posterior approach. Lenses were placed in a 24-well plate containing ATCC-formulated Eagle’s Minimum Essential Medium (EMEM, no. 30–2003, ATCC) supplemented with 10% fetal bovine serum (FBS, Corning 35–017-CV) and 1% penicillin/streptomycin/amphotericin (Gibco, Cat. No: 15140122). The plate was incubated at 37 °C under 5% CO_2_ for 2 h. After incubation, any visibly damaged or opaque lenses were discarded. The remaining intact lenses were then randomly allocated to appropriate experimental groups.

The NAC concentration was fixed at 5mM in all NAC-containing groups. Based on the drug loading capacity results, the amount of NAC-loaded GPT used in the NAC-GPT group was adjusted to ensure the same final NAC concentration (5mM). For the selenite-exposed groups, Na_2_SeO_3_ was added to corresponding media at a final concentration of 0.1mM, while the concentration of the other components remained unchanged. Lenses were incubated at 37 °C and 5% CO_2_ for 72 h. After incubation, lenses were gently rinsed with PBS and then carefully moved for imaging. The lenses were placed in a 12-well plate with a grid drawn on the bottom using a permanent marker to facilitate visual assessment of lens transparency. The lenses were maintained in 1 mL of phenol red-free EMEM during imaging. Images were acquired at 10× magnification using Leica software (Leica Microsystems Inc., Buffalo Grove, IL, USA) to provide an overall view of lens opacity. All lenses were graded according to the system described by Geraldine et al.,^24^ as follows: grade 0, no opacification (gridlines clearly visible); grade 1, slight opacification (minimal clouding of gridlines, yet still visible); grade 2, diffuse opacification involving almost the entire lens (moderate clouding of gridlines, gridlines faintly visible); and grade 3, extensive thick opacification involving the entire lens (total clouding of gridlines, gridlines not visible at all). Subsequently, lenses were immediately snap-frozen in microcentrifuge tubes and stored at −80 °C for analysis.^25^ The compositions of the treatment media and group abbreviations are summarized in Table 1.

**Table 1:** Treatment Media and Group Abbreviations for *Ex Vivo* Experiments

description (number of rat lenses)	incubation media	abbreviation
no treatment (*n* = 9)	cEMEM + PBS	PBS
NAC only (*n* = 9)	cEMEM + NAC	NAC
G5-PEG-TK only (*n* = 8)	cEMEM + GPT	GPT
NAC loaded G5-PEG-TK (*n* = 8)	cEMEM + NAC-GPT	NAC-GPT
selenite only (*n* = 6)	cEMEM + Na_2_SeO_3_	S
selenite + NAC (*n* = 6)	cEMEM + Na_2_SeO_3_ + NAC	S + NAC
selenite + G5-PEG-TK (*n* = 8)	cEMEM + Na_2_SeO_3_ + GPT	S + GPT
selenite + NAC loaded G5-PEG-TK (*n* = 7)	cEMEM + Na_2_SeO_3_ + NAC-GPT	S + NAC-GPT

### Irritation Test: Hen’s Egg Test on the Chorioallantoic Membrane (HET-CAM Test)

All experimental procedures were adopted from our prior study without substantial modification.^20^ For the irritation assessment, the formulation batch with the lowest drug-loading efficiency (LC% = 20%) was selected to ensure a consistent dosing concentration of 1 mg/mL NAC. Based on this loading efficiency, the corresponding concentrations of other components were calculated to be 4 mg/mL GPT and 5 mg/mL NAC-GPT.

### *In Vivo* Cornea Permeation and Biodistribution

SD rats were used and housed in conventional cages at the animal facility under a 12 h light/12 h dark cycle. All rat experiments were conducted in accordance with protocol 179–20, approved by the Institutional Animal Care and Use Committee. At 10 days of age, rat pups were divided into two primary groups (Figure 6A). One group underwent cataract modeling through intraperitoneal injection (IP) of Na_2_SeO_3_(19 μmol/kg), while the control group received an IP of PBS.^26^ The success of cataract modeling was confirmed using slit lamp examination in 15-day-old rats once their eyes had opened.^27^ Different formulations of eye drops were administered at 12 h intervals (morning and evening) with a volume of 5 μL per application. The corresponding treatment groups and abbreviations are presented in Table 2.

**Table 2:** Treatment Groups and Their Abbreviations for *In Vivo* Experiments Using the Normal and Cataract Models

normal model			
groups (number of rat eyes)	injection	eye drops (2 times/day)	abbreviation
no treatment (*n* = 9)	PBS	PBS	PBS
NAC only (*n* = 6)	PBS	NAC (1 mg/mL)	NAC-only
G5-PEG-TK only (*n* = 6)	PBS	GPT	GPT-only
NAC loaded G5-PEG-TK (*n* = 9)	PBS	NAC-GPT (NAC 1 mg/mL)	NAC-GPT

cataract model			
groups	injection	eye drops (2 times/day)	abbreviation
selenite only (*n* = 9)	Na_2_SeO_3_	PBS	PBS
selenite + NAC (*n* = 7)	Na_2_SeO_3_	NAC (1 mg/mL)	NAC-only
selenite + G5-PEG-TK (*n* = 7)	Na_2_SeO_3_	GPT	GPT-only
selenite + NAC loaded G5-PEG-TK (*n* = 9)	Na_2_SeO_3_	NAC-GPT (NAC 1 mg/mL)	NAC-GPT

Following 30 days of treatment, the rat lens morphologies were evaluated by using slit-lamp microscopy. Cataract severity was graded by a certified ophthalmologist (masked to the study) according to the following scale: grade 0—clear lens; grade 1—slight opacity; grade 2—partial nuclear opacity; and grade 3—dense nuclear opacity. After being graded, the rats were euthanized for sample collection. Following aqueous humor withdrawal, lenses were carefully dissected and imaged using the same imaging protocol as for an *ex vivo* lenses imaging. Aqueous humor (20 μL) was withdrawn by insulin syringe with fixed needle (Medline, 0.3 mL Syringe, 31G × 5/16 in. needle) for cornea permeation studies, and then 10 μL of aqueous humor was precipitated with 90 μL of methanol to remove proteins prior LC-MS analysis. LC-MS was used to quantify the drug concentrations in aqueous humor samples. Similarly, corneas and lenses were homogenized and had proteins removed before being analyzed by LC-MS.^28^

### Sample Preparation and MALDI-IMS Analysis

Enucleated eyeballs were rinsed with PBS and frozen in liquid nitrogen for biodistribution studies. The frozen tissues were embedded in 2% (w/v) carboxymethylcellulose (CMC) and stored at −80 °C prior cryosection.^29^ After equilibration in a cryostat at −20 °C, whole-eye blocks were sectioned at a thickness of 10 μm. Sections were mounted on 20 Ω indium tin oxide (ITO)–coated glass slides and coated with 1,5-diaminonaphthalene (DAN; Sigma-Aldrich) as the matrix.

Matrix-assisted laser desorption/ionization imaging mass spectrometry (MALDI-IMS) was performed using a Shimadzu LCMS-9030 iMScope QT system (Shimadzu, Kyoto, Japan)^30^ operated in positive-ion mode. Data acquisition and image reconstruction were controlled by iMScope QT LabSolutions software. Prior to analysis, instrumental parameters, including laser intensity, were optimized and kept constant throughout each imaging run. The MALDI laser was operated at a repetition rate of 2000 Hz with 200 laser shots per pixel, a laser diameter setting of 2, and a laser intensity setting of 60. The detector voltage was set to 2.2 kV, and ion images were acquired with a spatial pitch of 50 × 50 μm.

MALDI-IMS data analysis was performed using ImageReveal MS Mass Spectrometry Imaging Data Analysis Software (Shimadzu, Kyoto, Japan). Regions of interest (ROIs) corresponding to the lens area were manually defined based on the optical image and the corresponding ion images to ensure consistent anatomical localization across samples. Ion signals corresponding to NAC (*m*/*z* = 167.09), reduced GSH (*m*/*z* = 307.09), and oxidized GSSG (*m*/*z* = 612.09) were extracted from the defined ROIs. For each analyte, signal intensities within the ROIs were normalized to the total ion current (TIC) to minimize variability arising from matrix crystallization, laser fluctuation, or section thickness.

The TIC-normalized signal intensities were exported and further processed using GraphPad Prism (GraphPad Software, San Diego, CA, USA). Data were presented as bar graphs representing relative signal intensities, enabling a comparison of NAC, GSH, and GSSG distributions among different treatment groups. All MALDI-IMS analyses were performed in a qualitative and semiquantitative manner, and the extracted signal intensities were used to compare relative abundance rather than to determine absolute concentrations.

### Statistical Analysis

The plotting and statistical analysis were performed using GraphPad Prism 11. All results are expressed as the mean ± standard deviation of the mean (M ± SD). One-way ANOVA was used for testing statistical significance between groups. Statistical significance is indicated as follows: *p < 0.05, **p < 0.01, ***p < 0.001, and ****p < 0.0001.

## Results

### NAC Loading Efficiency and Characterization

The successful introduction of the thioketal linker was confirmed by ^1^H NMR (Figure S1). Compared with GP, GPT showed the appearance of the characteristic thioketal methyl resonance at ∼1.6 ppm, while retaining the PEG signal at ∼3.6 ppm and the G5 backbone peaks, supporting successful thioketal incorporation. Different NAC-loaded GPT nanogels were characterized by DLS for the size distribution measurements. After NAC loading, the samples were purified by centrifugation with ultracentrifuge tubes to remove the unloaded NAC and organic solvent. LC-MS measured the concentration of unloaded NAC for the EE% and LC% calculations. The formulations prepared with 0.8 mg/mL NAC exhibited a uniform size distribution with a polydispersity index (PDI) of 0.33, indicating good particle homogeneity and an average size of about 128 nm (Figure 2A). A loading capacity of 30% and an encapsulation efficiency of 57% were achieved when GPT was loaded with 0.8 mg/mL NAC (Figure 2B). Based on these results, we established a final ratio of 1 mg/mL of GPT to 0.8 mg/mL of NAC solution mixing at the same flow rate. The actual values of LC% and EE% were calculated for each preparation to ensure consistent NAC concentrations in NAC-GPT and NAC-only groups across all experiments.

**Figure 2:**
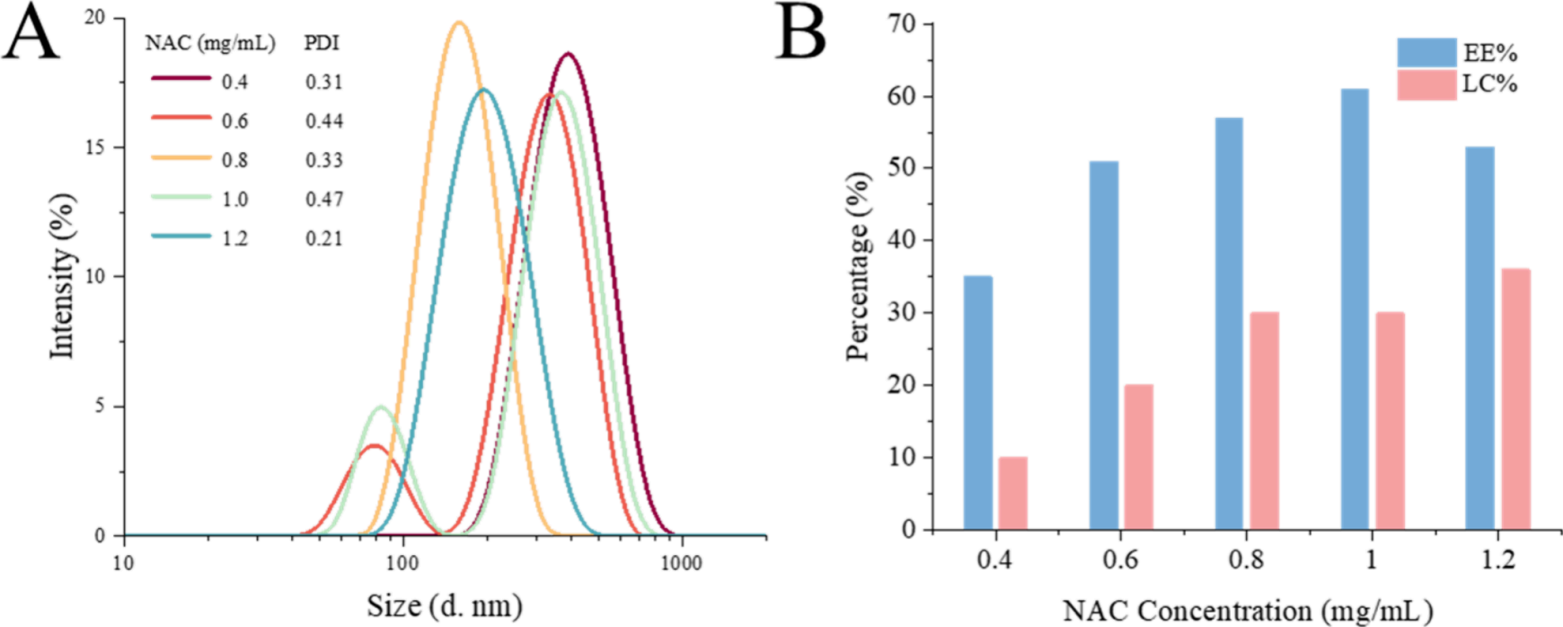
Particle size characterization and drug-loading efficiencies. (A) DLS size distributions and PDI value of prepared GPT with different NAC concentrations. (B) EE% and LC% of NAC-GPT synthesized with different NAC concentrations.

The morphologies of GPT and NAC-GPT nanogels were further characterized by TEM (Figure 3A,B). GPT nanogels were found to be uniform spheres with a size of about 80 nm (Figure 3A). Similarly, NAC-GPT nanogels displayed uniform morphology but were slightly larger with an average size of about 90 nm (Figure 3B). The surface charge of the formulations was evaluated by zeta potential measurements (Table S1). Compared with unmodified G5, PEGylation reduced the zeta potential to approximately +10.3 mV, consistent with partial shielding of surface charges by PEG chains. After thioketal cross-linking, GPT exhibited a higher apparent zeta potential of approximately +16.3 mV, which may reflect changes in nanogel architecture and interfacial charge presentation associated with network formation. Upon NAC loading, the zeta potential decreased to approximately +3.3 mV, consistent with partial charge neutralization by the anionic NAC. The flow behavior of the formulations was evaluated by steady-state shear rheology (Figure S2). All three samples exhibited shear-thinning behavior, with viscosity decreasing as shear rate increased, consistent with a non-Newtonian aqueous dispersion. Notably, GPT showed consistently higher viscosity and shear stress across the measured shear-rate range than G5 and GP, supporting the formation of a more cohesive nanoscale network after thioketal cross-linking. Despite this increase, the overall viscosity remained in a low range, suggesting favorable handling and instillation characteristics for topical administration.

**Figure 3:**
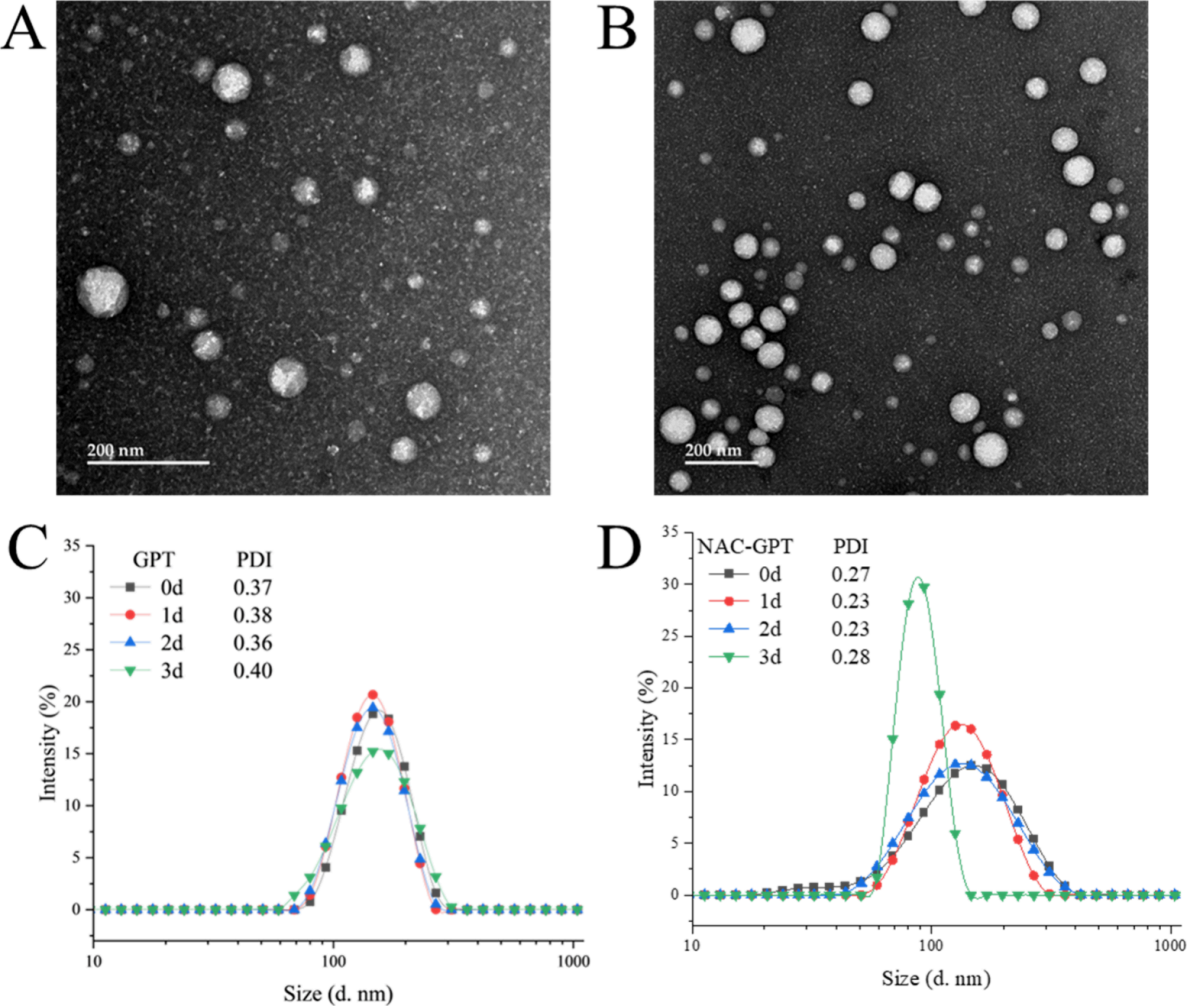
Morphology and stability of GPT and NAC-GPT (A, B). Representative TEM image of GPT (A), NAC-GPT (B), scale bar: 200 nm; (C, D) stability evaluation at different days of GPT (C) and NAC-GPT (D).

The larger size observed in DLS measurements of the fresh nanogels compared with the TEM results is a consequence of the drying process involved in TEM sample preparation. To evaluate stability, both formulations were suspended in PBS, and their size distribution was monitored at room temperature for 3 days. The DLS data indicated that GPT was stable throughout the three-day period at room temperature, with minimal changes in both size and PDI (Figure 3C). While the size distribution of NAC-GPT remained relatively stable during the initial 2 days, a slight decrease in size was noted on the third day, potentially due to the gradual release of NAC (Figure 3D). Notably, the PDI of NAC-GPT showed a minimal variation over the entire duration.

### *Ex Vivo* Evaluation of Antioxidant Effects in Rat Lenses

The *ex vivo* experiments aimed to assess whether the NAC-GPT could improve NAC uptake and antioxidant effects in whole lenses exposed to Na_2_SeO_3._ Na_2_SeO_3_, a well-established oxidative stress inducer, is commonly used for cataract modeling in both *ex vivo* and *in vivo* studies.^27^ *Ex vivo* models of Na_2_SeO_3_-induced cataracts have demonstrated its ability to alter lens morphology and disrupt the redox balance, as evidenced by alterations in GSH and GSSG levels. In this study (Figure 4A), rat lenses were divided into two main groups: a control group, where lenses were incubated with different formulations in standard culture medium without Na_2_SeO_3_, and an experimental group, where lenses were exposed to Na_2_SeO_3_ and the same formulations in the culture medium. This design enables comparative evaluation of lens morphology, NAC uptake, and redox-related response under static oxidative stress conditions.

**Figure 4:**
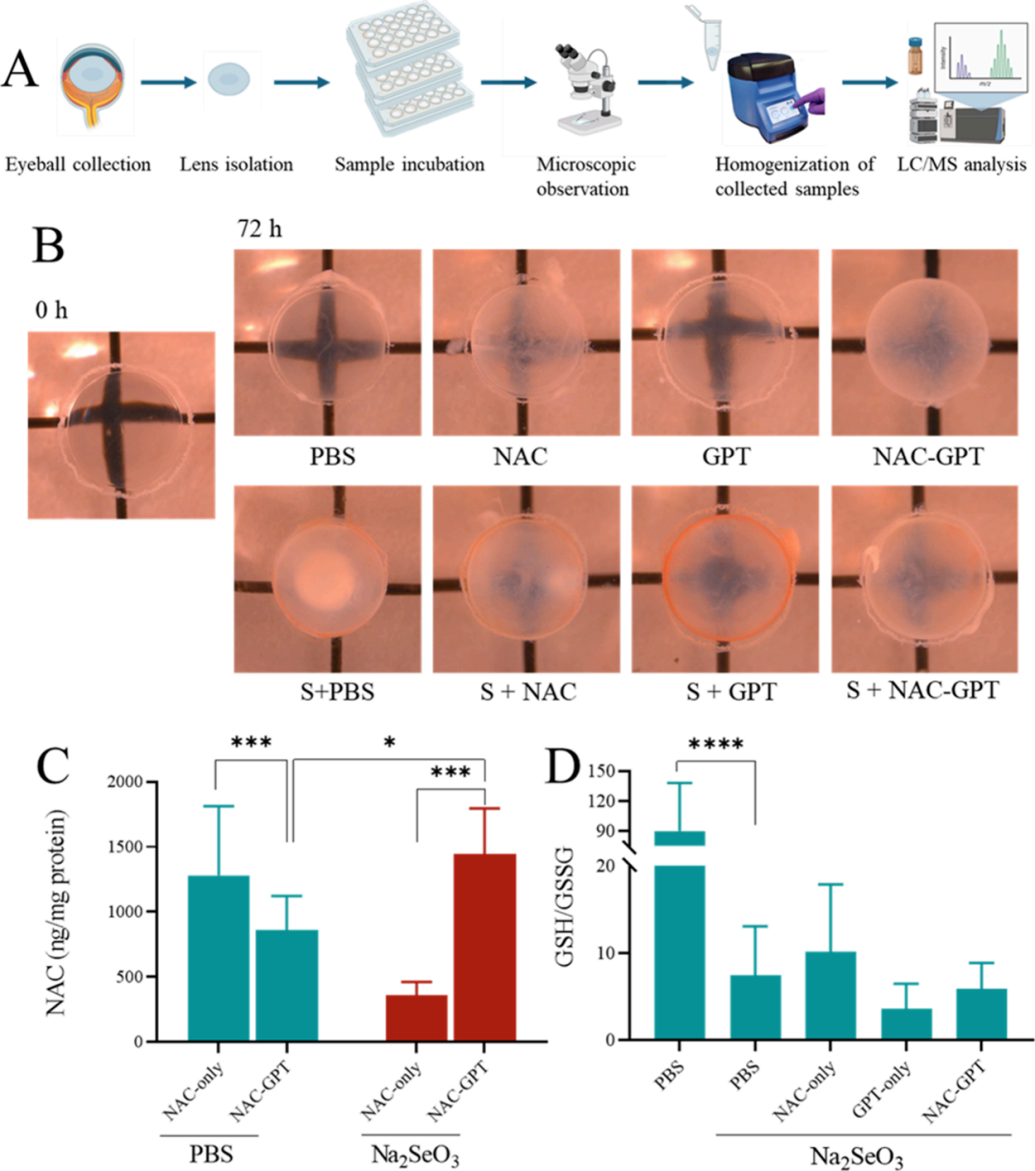
*Ex vivo* evaluation of antioxidant effects in rat lenses. (A) Schematic illustration of the *ex vivo* evaluation of antioxidant effects in rat lenses. (B) Representative morphological images of rat lenses at baseline (0 h) and after 72 h incubation with different formulations, illustrating the structural preservation and opacity changes. (C) Quantification of NAC concentrations in lenses following treatment with NAC-only and NAC-GPT formulations, measured via LC-MS, demonstrating the enhanced delivery efficiency of NAC-GPT. (D) GSH/GSSG ratio in lenses was assessed to evaluate oxidative stress levels after treatment with various formulations, demonstrating the antioxidative capacity of formulations. Data are presented as the mean ± SD (*n* = 6–8); *p < 0.05, ***p < 0.001, and ****p < 0.0001.

As shown in Figure 4B, morphological analysis clearly demonstrated the successful induction of cataracts by Na_2_SeO_3._ Following cataract severity grading across all groups (Table 3), lenses in Na_2_SeO_3_-only exhibited predominantly severe opacity (grade 3), whereas lenses from the PBS control group remained largely transparent (grade 0). Notably, cotreatment with antioxidant formulations was associated with reduced opacity under *ex vivo* conditions. Specifically, lenses cotreated with Na_2_SeO_3_ and antioxidant formulations (S+NAC, S+GPT, or S+NAC-GPT groups) display milder opacity, primarily ranging from grades 1 to 3, indicating a protective effect conferred by antioxidant formulations.

**Table 3:** Classification of Lens Opacity and Number of Cataracts Formed after 72 h of *Ex Vivo* Lens Culture

	treatment groups							
grading scale	PBS	NAC	GPT	NAC-GPT	S + PBS	S + NAC	S + GPT	S + NAC-GPT
grade 0	4	2	0	2	0	0	0	0
grade 1	1	1	3	4	1	1	3	0
grade 2	1	1	2	0	1	2	3	3
grade 3	0	1	1	0	5	2	1	2

Grading scale.

Grade 0—no opacification (gridlines clearly visible).

Grade 1—slight opacification (minimal clouding of gridlines; gridlines still visible).

Grade 2—diffuse opacification involving most of the lens (moderate clouding; gridlines faintly visible).

Grade 3—extensive opacification of the entire lens (total clouding; gridlines not visible).

Further assessment using LC-MS quantification of NAC concentrations in lens tissues (Figure 4C) demonstrated that, under normal condition, the lenses NAC concentrations in lenses were significantly higher in the NAC-only group (1281 ± 531.05) compared to the NAC-GPT group (858.71 ± 263.68, *p* < 0.001). In contrast, under oxidative stress conditions induced by Na_2_SeO_3,_ NAC-GPT group (1444 ± 396.77) exhibited significantly increased NAC accumulation compared to NAC-only (357.5 ± 103.04, *p* < 0.001). This enhanced accumulation suggests that encapsulation within the nanogel effectively protects NAC from oxidative degradation, thereby improving its bioavailability in the lens.

The GSH/GSSG ratio is a well-recognized indicator of oxidative stress and redox status. Its assessment is particularly relevant in the lens, where protein-rich structures are maintained in a reduced state largely by GSH. Therefore, we measured the GSH/GSSG ratio in lens samples to evaluate oxidative status.^26^ As shown in Figure 4C, lenses exposed to Na_2_SeO_3_-only (7.51 ± 5.60) had a significantly reduced ratio compared to the PBS group (90.23 ± 48.27, *p* < 0.0001), indicating elevated oxidative stress (Figure 4D). Although the S + NAC group exhibited a tendency to increase the GSH/GSSG ratio, the difference was not statistically significant compared to that of the Na_2_SeO_3_-only group (Figure 4D). These findings suggest that while NAC uptake is enhanced by the nanogel under oxidative conditions, the restoration of redox balance is limited in the *ex vivo* lens model.

Prior to the *in vivo* rat experiments, a HET-CAM assay was performed to evaluate the mucosal irritation potential of each formulation and to minimize the risk of adverse effects in subsequent animal studies. A 20 μL sample of each formulation was applied to the CAM surface, and the membrane was observed over 5 min for any signs of irritation (hemorrhage, coagulation, or lysis). No visible signs of irritation or vascular damage were detected in the membranes treated with any formulation, except NaOH, a positive control, during this observation period (Figure 5). As summarized in Table 4, none of the tested formulations, including the 1 mg/mL NAC group, caused any significant irritation to the CAM. These results support the safety of the tested formulations, thereby justifying their use in subsequent *in vivo* studies.

**Figure 5:**
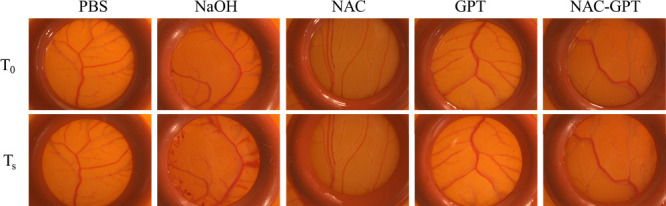
Irritation evaluation using the hen’s egg test-chorioallantois membrane assay. Stereomicroscopic images of the CAM vasculature before treatment (T_0_) and 5 min after treatment (Ts) with 1 mg/mL NAC, 4 mg/mL GPT, and 5 mg/mL NAC-GPT, compared with 1 M NaOH (positive control) and PBS (negative control). The dosing volume was 20 μL (*n* = 3).

**Table 4:** Irritation Scores of the HET-CAM Test

sample	NaOH (1 M)	control (PBS)	NAC (1 mg/mL)	GPT (5 mg/mL)	NAC-GPT (5 mg/mL)
irritation score (IS)	18.6	0.07	0.07	0.07	0.07
irritation category	strong irritation	no irritation	no irritation	no irritation	no irritation

The score range: 0–0.09, nonirritant; 0.1–4.9, weak or slight irritation; 5–8.9 or 5–9.9, moderate irritation; and 9–21 or 10–21 strong or severe irritation.

### *In Vivo* Distribution of NAC and Evaluation of Antioxidant Effects in Rat Lenses

Intraperitoneal injection of Na_2_SeO_3_ (19 μmol/kg) on day 10 was sufficient to induce cataract formation in rat pups. For the control groups, another set of rat pups was injected with an equivalent volume of PBS intraperitoneally. By the time the pups opened their eyes at 15-day-old, cataract formation was visibly evident. This was further confirmed through examination using a slit-lamp microscope. After eye opening, both groups (cataract and normal) were treated with different formulations twice daily for 30 consecutive days (Figure 6A). After treatment, lens conditions were imaged by slit-lamp imaging, followed by dissection and direct imaging of excised lenses (Figure 6B,C). Cataract severity was assessed by using a standardized scoring system based on morphological changes following treatment. In the normal PBS group, 70% of rats presented with clear lenses (grade 0), whereas in the cataract PBS group, approximately 70% of animals exhibited advanced cataracts (grade 2 or 3), as summarized in Table 5.

**Figure 6:**
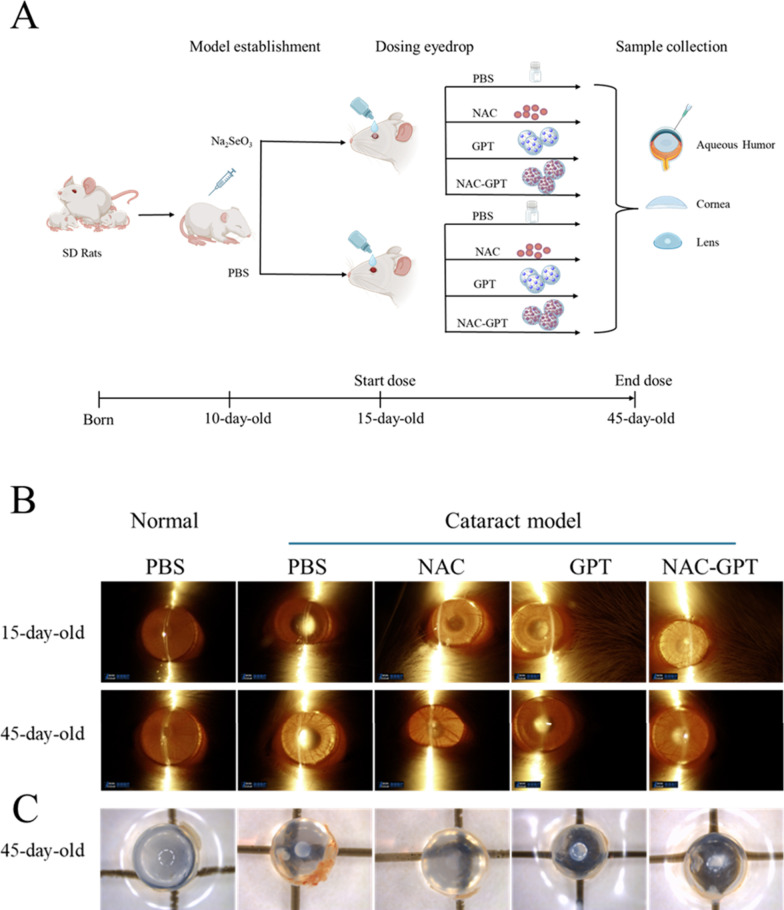
*In vivo* evaluations of treatment efficacy in normal and cataract models. (A) Schematic representation of the experimental timeline and treatment protocol for normal and cataract-induced models. (B) Slit lamp images showing progression in lens clarity for untreated (PBS) and treated eyes (NAC-GPT; GPT and NAC) in the cataract model, captured at 15-day-old (before treatment) and 45-day-old (after treatment for 30 days). (C) Postsacrifice, images of lenses dissected from ocular, highlighting visual differences in lens opacity across different groups.

**Table 5:** Classification of Lens Opacity and Number of Cataracts Observed in Rat Lenses Following 30 Days of Treatment

	treatment groups							
	normal model				cataract model			
grading scale	PBS	NAC	GPT	NAC-GPT	PBS	NAC	GPT	NAC-GPT
grade 0	6	3	2	3	1	1	1	0
grade 1	1	1	1	4	2	0	1	4
grade 2	1	0	1	1	2	2	2	2
grade 3	1	3	1	1	4	4	3	3

Grading scale.

Grade 0—clear lens.

Grade 1—slight opacity;

Grade 2—partial nuclear opacity;

Grade 3—dense nuclear opacity.

To evaluate the intraocular drug distribution, NAC concentrations in the cornea, aqueous humor, and lens were quantified using LC-MS (Figure 7). The NAC concentration in the cornea did not show statistically significant differences among the groups. However, the NAC-GPT group exhibited a trend toward higher NAC levels compared with the NAC-only group (Figure 7A). In the aqueous humor, the NAC concentration in the normal group treated with NAC-GPT (0.91 ± 0.25 ng/mg protein) was significantly higher than that in the NAC-only group (0.49 ± 0.23 ng/mg protein, *p* < 0.01), indicating enhanced retention of NAC by the nanogel system. In the cataract model, there was no significant difference in NAC concentrations in the aqueous humor between the NAC-GPT and the NAC-only group (Figure 7B). Notably, the NAC concentration in the lens was significantly higher level in the NAC-GPT group (0.44 ± 0.12 ng/mg protein) compared to the NAC-only group in the normal model (0.12 ± 0.06 ng/mg protein, *p* < 0.0001). Similarly in the cataract model, the NAC-GPT group (0.21 ± 0.08 ng/mg protein) also showed significantly higher than the NAC-only group (0.09 ± 0.04 ng/mg protein, *p* < 0.05), demonstrating that the nanogel system effectively enhances drug delivery to the lens (Figure 7C). To further visualize the NAC distribution in ocular tissues, MALDI-TOF was performed on whole-eye cryosections (Figure 7D). MALDI imaging further suggested a cortex-dominant distribution pattern, consistent with known intralenticular transport compartmentalization. Semiquantitative NAC signal intensities across the whole eye section (Figure 7E) showed an overall higher trend in the NAC-GPT group compared to the NAC-only group, highlighting the enhanced ocular retention and bioavailability achieved by the nanogel formulation.

**Figure 7:**
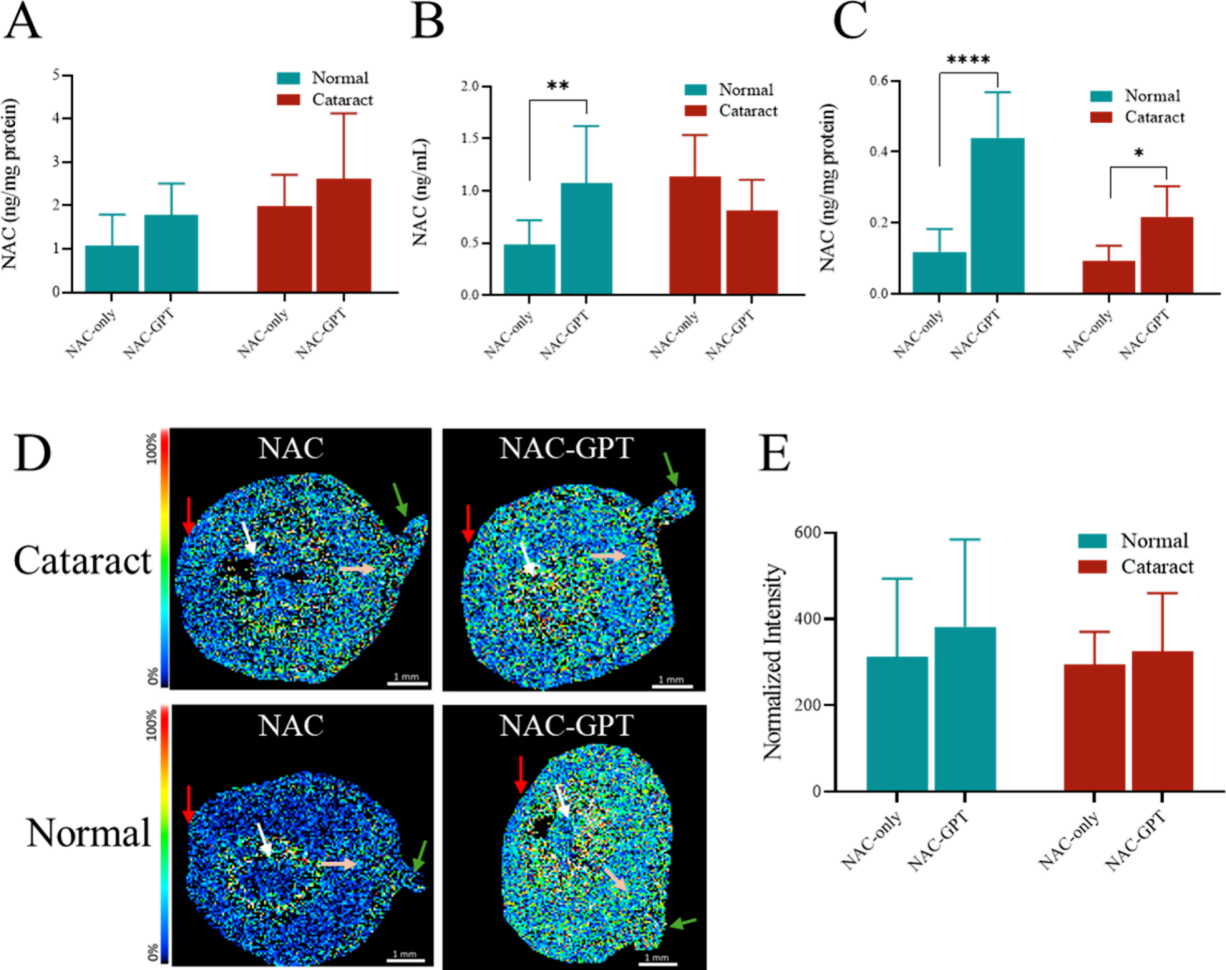
*In vivo* quantification of NAC concentrations in ocular tissues. (A–C) NAC concentrations via LC-MS in various ocular tissues, including the cornea (A), aqueous humor (B), and lens (C), following treatments with NAC-only and NAC-GPT formulations in both normal and cataract models. (D) Representative MALDI-TOF illustrates the spatial distribution of NAC in whole eye cryosections from rats treated with NAC-GPT and NAC-only (red arrow: cornea, white arrow: lens, pink arrow: posterior part of eye, green arrow: optic nerve. *n* = 3). (E) Quantitative analysis of NAC signal intensities obtained from MALDI-TOF images across the entire ocular section. Data are presented as the mean ± SD (*n* = 7–9); *p < 0.05, **p < 0.01, and ****p < 0.0001.

To assess the therapeutic antioxidant effect of the NAC-GPT formulation, we measured the GSH/GSSG ratio in the lens from both the cataract model and normal rats after 30 days of treatment. In the cataract group (Figure 8A), the GSH/GSSG ratio demonstrated significant elevation exclusively in the NAC-GPT-treated group (58.30 ± 16.93) when compared to the group treated with PBS (35.05 ± 4.22, *p* < 0.01). This indicates the capability of the NAC-GPT formulation to restore redox balance and mitigate oxidative stress in the lens under cataract-inducing conditions. In the normal rat model (Figure 8B), GSH/GSSG ratios remained relatively consistent across all groups, suggesting that the therapeutic antioxidant effect of NAC-GPT is particularly prominent under oxidative stress conditions. To visualize the GSH and GSSG distribution in cataract model lenses, MALDI-TOF imaging was performed on whole-eye cryosections (Figure 8C). The NAC-GPT-treated lenses exhibited a higher GSH signal intensity (Figure 8D), reaching approximately 5000 arbitrary units (au), which was about 1.5 times greater than the ∼3026 au observed in PBS-treated selenite-induced cataract group. In contrast to GSH, GSSG signals were considerably low, with no significant differences among groups, showing intensities close to background levels (Figure 8E). These results confirm that NAC-GPT not only enhances intraocular NAC delivery but also exhibits potent antioxidant effects in vivo, effectively restoring redox homeostasis and reducing the oxidative stress-induced opacity in the lens.

**Figure 8:**
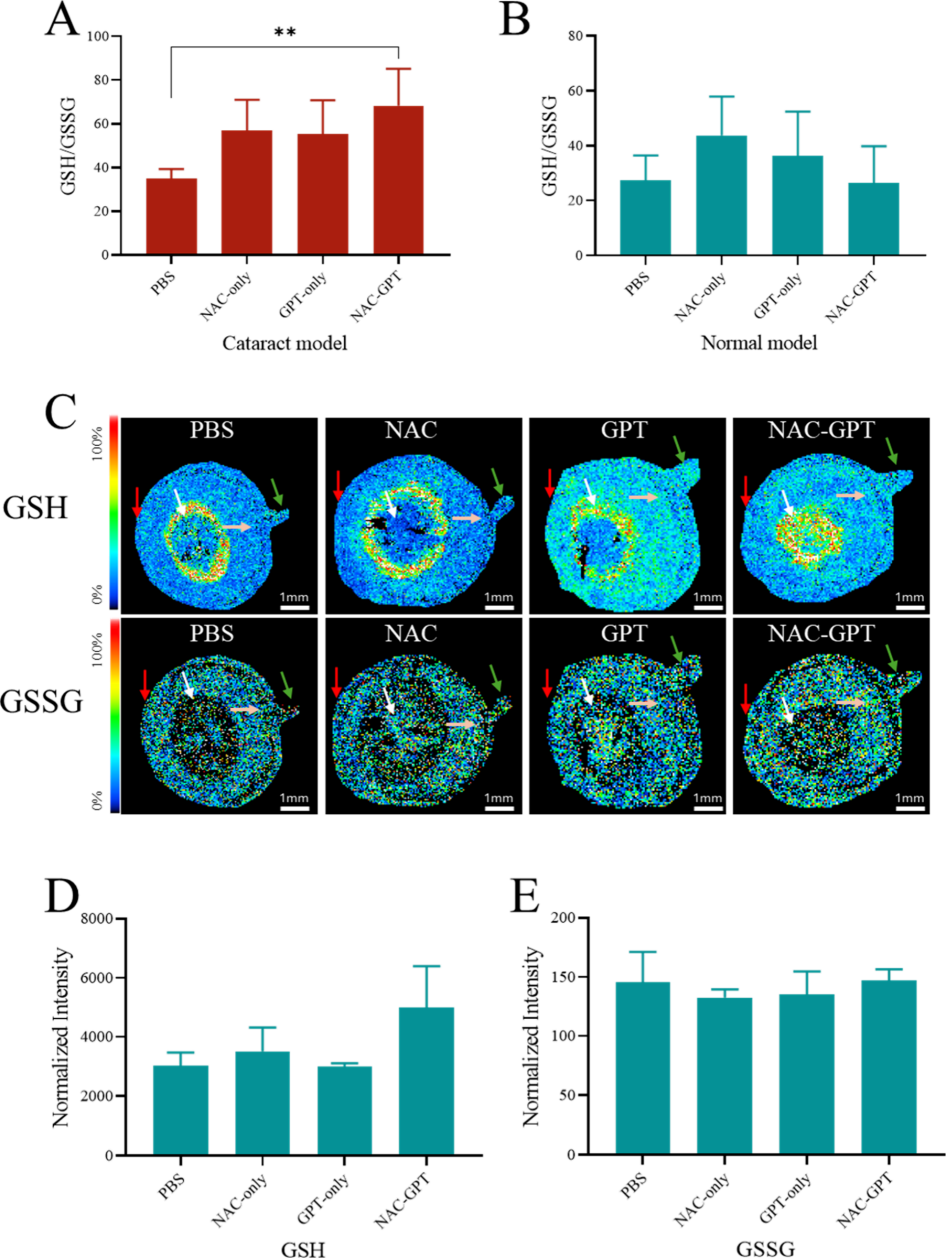
*In vivo* evaluation of antioxidant effects of different formulations in rat lens. (A, B) GSH/GSSG ratios measured in rat lenses from the cataract model (A) and normal rats (B) following treatment with different formulations. (C) MALDI-TOF ion image showing the spatial distribution of GSH and GSSG in the whole eye cryosections from cataract model group after treatment (red arrow: cornea, white arrow: lens, pin arrow: posterior part of eye, green arrow: optic nerve; *n* = 3). (D, E) Quantitative analysis of GSH (D) and GSSG (E) signal intensity in the lens region based on MALDI-TOF data. Data are presented as the mean ± SD (*n* = 7–9); **p < 0.01.

## Discussion

Dendrimers have long been recognized as promising carriers for drug delivery due to their highly branched structure, numerous surface functional groups, and nanoscale dimensions.^19^ These characteristics are particularly advantageous for ocular drug delivery, where challenges, such as poor drug permeability, rapid clearance, and ocular barriers, significantly limit therapeutic efficacy. In our previous work, we developed ROS-scavenging nanogels by cross-linking G5 dendrimers with a ROS-responsive linker, TK-NHS, via flash nanoprecipitation.^20^ This MIVM-based method enabled the rapid, reproducible, and scalable formation of stable and uniform nanogels. The resulting nanogels exhibited ROS-scavenging capacity, making them a promising delivery system for therapeutics targeting oxidative stress–related ocular diseases.^8^

Based on the promising characteristics of our previously developed ROS-scavenging nanogels, the present study explores their potential for enhanced ocular delivery of antioxidant drugs. To improve the colloidal stability and bioadhesiveness of the nanogels, G5 dendrimer was functionalized with PEG, resulting in a PEGylated nanogel structure (GPT).^31^ Importantly, we utilized the rapid mixing and cross-linking capability of MIVM to encapsulate the drug directly during nanogel formation.^32^ This method not only simplified the preparation process but also improved the drug-loading efficiency. The PEGylated nanogels maintained a uniform size distribution, and a slight increase in their hydrodynamic diameter following drug loading confirmed successful encapsulation of the drug without compromising their stability.^33,34^

As a model drug in this study, NAC, a well-known antioxidant, has been previously studied for its therapeutic potential in ocular diseases.^35^ However, its clinical translation has been hindered by poor bioavailability and rapid clearance. The lens is an avascular tissue located in the center of the eye and is shielded by multiple ocular barriers that limit drug penetration. Because the lens lacks a direct blood supply, systemic delivery provides limited exposure, making topical administration one of the most practical routes for delivering antioxidants to the lens. Furthermore, topically applied small-molecule antioxidants such as NAC are prone to rapid metabolism and elimination. In addition, the conventional passive diffusion-loading methods can rarely obtain a high loading capacity for hydrophilic drugs, such as NAC. To address these challenges, our nanogel system was developed to enhance corneal permeability and to support a more reducing microenvironment in the lens by improving antioxidant availability. By helping to maintain GSH in its reduced form, this strategy is expected to mitigate oxidative modification of lens proteins, including disulfide bond formation and crystallin aggregation, which are closely associated with lens opacification.

Our *ex vivo* experiments demonstrated that NAC-GPT significantly increased NAC accumulation in lenses exposed to Na_2_SeO_3_-induced oxidative stress. In contrast, the NAC-only group showed minimal NAC retention, highlighting the protective and delivery-enhancing role of the nanogel under oxidative conditions. In the *in vivo* study, no statistically significant NAC concentration differences were observed in the cornea and aqueous humor samples among the treatment groups. However, the cumulative NAC levels in the lenses were markedly higher in the NAC-GPT group compared to the NAC-only group, with approximately a 2-fold increase observed in both normal and cataract model rats. Despite this increased whole-lens NAC exposure in the cataract model, slit-lamp evaluation did not show an evident reduction in nuclear opacity. In agreement with this observation, MALDI imaging showed relatively low NAC signal intensity in the lens nucleus compared with peripheral lens regions. These results suggest that the nanogel formulation improves NAC transport across the cornea and into the aqueous humor and enhances delivery to the lens, but intralenticular diffusion barriers may restrict distribution toward the nucleus and may therefore limit the extent to which topically delivered NAC influences nuclear opacity in this model.^36–38^

The *ex vivo* experiments demonstrated that under normal conditions, the lens NAC concentration was significantly higher in the NAC-only group than in the NAC-GPT group. However, under oxidative conditions induced by Na_2_SeO_3_, the trend was reversed, with the NAC-GPT group showing significantly higher lens NAC levels. As is known, Na_2_SeO_3_ is a redox-active inorganic selenium compound that can potentially react with thiol-containing reductants such as NAC, forming reduced selenium intermediates or covalent selenium–sulfur adducts and thereby consuming free NAC.^39,40^ This suggests that free NAC is highly susceptible to oxidative degradation under high oxidative stress conditions, whereas nanogel encapsulation of NAC preserves it during transit to the lens.

Furthermore, analysis of redox status revealed a significant increase in the GSH/GSSG ratio exclusively in the *in vivo* cataract model treated with NAC-GPT, compared to PBS controls, confirming improved redox regulation under oxidative stress conditions. To further visualize the distribution of GSH species directly and to account for potential sample loss during tissue processing, we also performed MALDI imaging on the whole eye cryosections. In this study, MALDI imaging was used to provide spatial visualization and semiquantitative comparison based on normalized signal intensities, whereas LC-MS served as the primary method for absolute quantification from whole-lens homogenates. The MALDI imaging results were consistent with LC-MS data, showing an approximately 1.5-fold higher GSH signal in the NAC-GPT group compared to the PBS-treated group. Although GSSG signals were close to background levels and did not exhibit clear differences, the consistent GSH trends observed with both MALDI imaging and LC-MS support the reliability of our findings. Together, these results highlight that restoration of redox balance was evident *in vivo*, whereas the *ex vivo* model is limited in capturing long-term redox homeostasis due to the absence of physiological antioxidant synthesis and recycling pathways.

Importantly, in addition to ocular surface and anterior segment barriers, intralenticular and transport limitations can further restrict the delivery of antioxidants to morphologically and functionally distinct regions of the lens. Previous studies have reported pronounced spatial gradients of GSH, with higher levels in the cortex and lower levels in the nucleus, reflecting regional differences in metabolism and transport.^41^ The lens is an avascular tissue that grows throughout life by adding new fiber cell layers at the cortex, creating structurally distinct compartments. The outer cortex contains differentiating, metabolically active fiber cells that retain the capacity for *de novo* GSH synthesis, whereas the nuclear region comprises long-lived mature fiber cells that have lost their organelles and possess limited biosynthetic capacity. This compartmentalized architecture, together with restricted molecular transport within the densely packed fiber mass, contributes to the well-documented cortex-to-nucleus GSH gradient.^15^ Consistent with these structural features, our MALDI imaging revealed a cortex-to-core gradient of the GSH signal. The NAC signal was predominantly localized to the lens cortex, suggesting that further optimization is needed to enhance delivery to the lens nucleus. This is particularly relevant because the selenite model produces a predominantly nuclear cataract phenotype that resembles key features of age-related nuclear cataract. Accordingly, our LC-MS measurements represent whole-lens averaged values, whereas MALDI provides a complementary spatial context. Together, these results support enhanced ocular delivery and improved redox regulation *in vivo*.

In summary, our data demonstrate that NAC-GPT enhances intraocular NAC delivery and improves redox regulation *in vivo* under cataract-inducing conditions. This multifunctional nanogel platform improves drug stability and bioavailability and represents a promising noninvasive antioxidant delivery strategy for ocular applications involving oxidative stress.

## Conclusions

In this study, we successfully developed a multifunctional PEGylated dendrimer nanogel (NAC-GPT) for effective ocular NAC delivery. Utilizing a rapid and scalable flash nanoprecipitation strategy via MIVM allowed for the production of highly uniform nanogels with efficient hydrophilic drug encapsulation. The integration of a ROS-responsive thioketal linker endowed the nanogels with strong antioxidative properties, enabling them to protect encapsulated NAC from oxidative degradation and controlled release in high ROS microenvironments.

Our comprehensive *ex vivo* and *in vivo* experiments confirmed that the NAC-GPT system significantly improved NAC penetration and retention in ocular tissues, especially under cataract-inducing oxidative stress. Importantly, improved redox regulation, as evidenced by increased GSH/GSSG ratios and enhanced GSH signal intensity, was observed specifically in the *in vivo* cataract model. These findings indicate that the GPT nanogel platform can effectively deliver thiol compounds to the eye and represents a promising noninvasive antioxidant delivery strategy with the potential to help maintain redox balance in cataract-affected lenses.

## Supplementary Material



## Notes

Rheometer testing protocol; zeta potentials of G5, GP, GPT, and NAC-GPT formulations; ^1^H NMR spectra of G5-PEG and G5-PEG-TK in D_2_O; and rheological characterization of G5, GP, and GPT (5% w/v), viscosity and shear stress vs shear rate at 37 °C (PDF)

## Abbreviations

ROS: reactive oxygen species
NAC: N-acetylcysteine
GPT: G5-PEG-TK (generation-5 PEGylated poly­(amidoamine) dendrimer nanogel)
GSH: glutathione
GSSG: oxidized glutathione
LC-MS: liquid chromatography–mass spectrometry
MALDI-TOF: matrix-assisted laser desorption/ionization time-of-flight
TEM: transmission electron microscopy
DLS: dynamic light scattering
EE%: encapsulation efficiency
LC%: loading capacity
IP: intraperitoneal injection
MIVM: multi-inlet vortex mixer
PDI: polydispersity index
