# Supplementary material for: Dendrimer Nanogels with Built-in Free Radical Scavenging Enable Efficient Topical Delivery of a Hydrophilic Antioxidant to Restore Lens Redox Balance for Cataract Treatment
Source: ACS Appl Mater Interfaces. 2026 Mar 19;18(12):17482–94. doi: 10.1021/acsami.5c24072 (PMC13051436; doi:10.1021/acsami.5c24072)
Supplement: Supplementary file 1 [file am5c24072_si_001.pdf]

Supporting Information

Dendrimer Nanogels with Built-in Free Radical Scavenging Enable Efficient Topical Delivery of a Hydrophilic Antioxidant to Restore Lens Redox Balance for Cataract Treatment

Lin Qi[1], Huari Kou[2], Anna Chernatynskaya[2], Da Huang[3], Vimalin Jeyalatha Mani[1], Humeyra Karacal[4], Nuran Ercal[5,*], Hu Yang[1,*]

[1]Joint Department of Biomedical Engineering, Marquette University and Medical College of Wisconsin, Milwaukee, WI 53226, United States

[2]Linda and Bipin Doshi Department of Chemical and Biochemical Engineering, Missouri University of Science and Technology, Rolla, MO 65409, United States

[3]College of Biological Science and Engineering, Fuzhou University, Fuzhou, Fujian 350108, China

[4]Premiere Eye Associates, 816 S. Kirkwood, St. Louis, MO 63122, United States

[5]Department of Chemistry, Missouri University of Science and Technology, Rolla, MO 65409, United States

*Corresponding authors: nercal@mst.edu (NE) and huyang@mcw.edu (HY)

Rheometer Testing.

Lyophilized G5, GP, and GPT samples were stored as powder and reconstituted in deionized water to prepare 5% (w/v) solutions prior to testing. Rheological measurements were performed using a TA Instruments hybrid rheometer (TA Instruments, New Castle, DE, USA) equipped with a Peltier temperature-control system. Samples were tested using a 40 mm stainless-steel parallel-plate geometry with a 1000 μm gap (minimum sample volume ~1.26 mL). All measurements were conducted at 37 °C following a 180 s equilibration period. A logarithmic flow sweep was performed over a shear-rate range of 0.1–1000 s^{-1} with 5 points per decade. Steady-state sensing was enabled with a maximum equilibration time of 90 s, a sample period of 10 s, a 5% tolerance criterion, and a requirement of three consecutive readings within tolerance before recording each data point.

Table S1. Zeta potentials of formulations

Sample	ζ- Potential (mV)
G5	17.3
GP	10.3
GPT	16.3
NAC-GPT	3.3

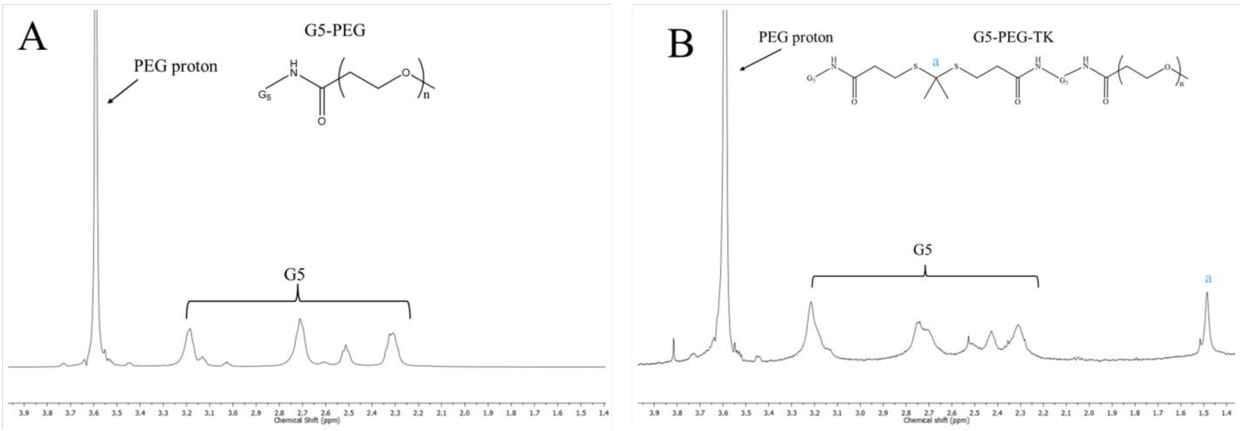

Figure S1. ^{1}H NMR spectra of G5-PEG and G5-PEG-TK in D_2O.

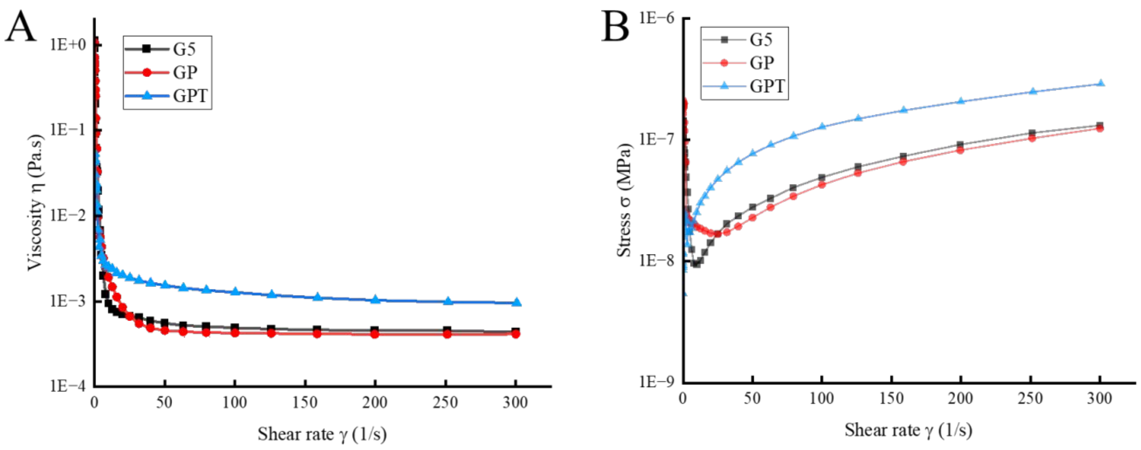

Figure S2. Rheological characterization of G5, GP, and GPT (5% w/v in deionized water) showing viscosity (A) and shear stress (B) as a function of shear rate, measured at 37 °C.
